# Society Meetings

**Published:** 1918-01

**Authors:** 


### SOCIETY MEETINGS

**The Buffalo Academy of Medicine** passed the following resolution at a council meeting on October 5th: "Dues of members in military service of U. S. Army or Red Cross be omitted and that members be considered in good standing until return to civil practice; and that any moneys paid by them while in active service be refunded." Notify Secretary on entrance to service.

---

**Buffalo Academy of Medicine.** Section of Obstetrics and Gynecology, Wednesday, November 21, program: Conservative Treatment of Eclampsia, by Dr. R. McPherson, New York City.

Section of Obstetrics and Gynecology, Wednesday, November 28, Program: The Micro-organisms of the Mouth and Throat and their Significance, by Dr. O. W. Mitchell, Syracuse, N. Y. Discussion opened by Dr. DeLancy Rochester.

Surgical Section: Wednesday, December 5, Program: Non-union in fractures Resulting from Gunshot Wounds, by Dr. W. E. Gallie, Toronto, Ont. Discussion opened by Dr. E. R. McGuire. The Place of Intra-Spinous Therapy in Urology, by Dr. E. M. Watson, Buffalo.

Section of Medicine, Wednesday, December 12. Program: The Drug Habit: Alcoholism, Morphinism, Nicotinism, by Dr. Henry R. Hopkins. Some Interesting Cases of General Infections from Local Foci, by Dr. DeLancy Rochester.

Section of Obstetrics and Gynecology, Wednesday, Decem-19th. Program: Delivery by Foot or Feet, Dr. P. W. Van Peyma; The Nasal Reflex in Gynecology, Dr. G. F. Cott.

---

**Elmira Academy of Medicine,** Wednesday, December 5. Annual election of officers. Program: President's Address; Dr. R. B. Howland, Dr. F. L. Christian.

---

**Medical Society of the State of New York.** Section on Pediatrics. The following doctors participated in the annual fall clinical day, December 13th, New York City: Clinic on the Treatment of Diabetes in Children, by Dr. Frederick M. Allen; New Method of Estimating Acidosis in the Blood, by Dr. Donald Van Slyke; Clinic on Newer Methods of Diagnosis and Treatment of Hydrocephalus, by Dr. Chas. A. Elsberg; The Surgical Treatment of Birth Palsy, by Dr. Alfred S. Taylor; Practical Demonstrations of Some Recent Clinical Methods, by Drs. Linneaus La Fetra, Chas. H. Smith, Oscar M. Schloss, Herbert B. Wilcox; Arthritis of Scarlet Fever, by Dr. C. P. Sharpe; New Plans for the Health Centers, by Dr.

Haven Emerson; The National Danger from Defective Development of Growing Children in Time of War, by Dr. Henry D. Chapin; The Hypertonic Infant, the Curative Action of Atropin upon Certain of its Manifestations, by Dr. Sidney V. Haas.

---

**Rochester Academy of Medicine.** Meeting of Section IV. Wednesday, December 12. Program: War, and Its Effects on the Civil Population During and After War, Dr. George Goler.

---

**Medical Society of the County of Erie.** Annual election December 17: President, Dr. George F. Cott; 1st Vice-President, Dr. James E. King; 2d Vice-President, Dr. Earl P. Lothrop; Secretary, Dr. Franklin C. Gram; Treasurer, Dr. Albert T. Lytle; Censors, Dr. John D. Bonnar, Dr. Francis C. Fronczak, Dr. Arthur G. Bennett, Dr. Archibald D. Carpenter, Dr. Frank A. Valente; Chairman Committee on Legislation, Dr. Harvey R. Gaylord; Chairman Committee on Public Health, Dr. Nelson G. Russell; Chairman Committee on Membership, Dr. William F. Jacobs; Chairman Committee on Economics, Dr. John V. Woodruff; Delegates to State Society, Dr. Arthur G. Bennett, Dr. George F. Cott, Dr. Julius Richter, Dr. Franklin W. Barrows.

The Government requires each county to furnish 15 per cent of all practicing physicians for the Medical Reserve Corps. Erie County has thus far furnished less than 10 per cent and efforts are being made to secure the remainder. Immediate volunteers are requested.

